# Fiat Lux: Light and Pedagogy for the 21st Century

**DOI:** 10.1177/09727531221136646

**Published:** 2023-05-19

**Authors:** James C. Lech, Matthew T.J. Halma, Adejoke O. Obajuluwa, Malcolm Baker, Michael R. Hamblin

**Affiliations:** * These authors share joint first authorship.; † Passed away June 16, 2021; 1 Vrije Universiteit Amsterdam, De Boelelaan, Amsterdam, The Netherlands; 2 Department of Radiology and Nuclear Medicine, Academic Medical Center, University of Amsterdam (UMC), Amsterdam, The Netherlands; 3 National Research Foundation, South Africa; 4 International EMF Project & Optical Radiation, World Health Organization, Pretoria, South Africa; 5 Biotechnology Unit, Department of Biological Sciences, Afe Babalola University, Olusegun Obasanjo Way, Ado Ekiti, Nigeria; 6 Department of Neurology, 1 Military Hospital, Pretoria, Department of Defence, South Africa Military Health Service Pretoria; 7 Department of Neurology, University of Pretoria, South Africa; 8 Laser Research Centre, Faculty of Health Science, University of Johannesburg, Doornfontein, South Africa

**Keywords:** Pedagogy, built environment, biophilic design, photobiomodulation, light therapy, educational design

## Abstract

**Background:**

The relationship between the quality of the learning environment and student outcomes is receiving more serious attention from educational psychologists, neurologists, ophthalmologists, orthopedists, surgeons, oncologists, architects, ergonomists, nutritionists, and Michelin star chefs. There is a role for ergonomic office and school design to positively impact worker and student productivity, and one design attribute drawing attention is the indoor lit environment. In this review, we expand upon the role that light plays in education, as it has enabled millions of pupils to read at late hours, which were previously too dark. However, still unappreciated is the biological effects of artificial light on circadian rhythm and its subsequent impacts on health and learning outcomes.

**Summary:**

This review describes the current state of light in the educational environment, its impact, and the effect of certain inexpensive and easy-to-implement adaptations to better support student growth, learning and development. We find that the current lighting environment for pupils is sub-optima based on biological mechanism and may be improved through cost effective interventions. These interventions can achieve greater biological harmonization and improve learner outcomes.

**Key Message:**

The impact of the lighting environment in educational institutions on pupil biology has received minimal attention thus far. The current lighting environment in schools is not conducive to student health and educational performance. Cost-effective approaches can have an outsized impact on student health and educational attainment. We strongly recommend educational institutions take the lit environment into account when designing educational programs.

## Introduction

**Figure 1.**
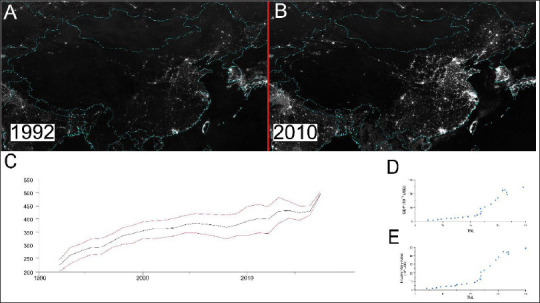
Electric Light Has Grown Significantly in the Last Quarter-Century, with Economic Benefits.

**Note:** (A) Nighttime satellite imagery of China in 1992 and (B) a similar photo shown in 2017, a 25-year span. Significant growth of electric lighting has accompanied economic development. Satellite images were taken from (https://eogdata.mines.edu/products/movie_loop/#dnb) under a CC-BY-SA license (https://creativecommons.org/licenses/by-sa/4.0/). (C) Global light intensity, measured as digital number (DN), a proxy for brightness, as a function of time. Adapted from.^2^ DN values are calculated from the datasets Defense Meteorological Satellite Program Operational Linescan System (DMSP-OLS) and the Suomi National Polar-Orbiting Partnership Visible Infrared Imaging Radiometer Suite (NPP-VIIRS) datasets. (D) Gross Domestic Product (GDP) (top) and Industrial Value Added (IVA) (bottom) in 10^11^ USD as a function of Total Night Light (TNL). Adapted from Hu and Zhang.^2^

Electric lighting, since its development in the late 1800s, has revolutionised modern life, allowing humans to operate at hours not normally available for work. Electric lighting has increased significantly over the past 25 years, especially in previously less developed nations (Figure 1A and B). Globally, the use of electric light almost doubled from 1992 to 2017 (Figure 1C). While electrification has contributed to broad economic prosperity and convenience (a correlation between total night light (TNL) and China’s gross domestic product (GDP) is shown in Figure 1D, and a correlation between TNL and industrial value added (IVA) is shown in Figure 1E), there has been a significant biological cost to individuals who deviate from our natural circadian cycles.^1^ Developments such as electric light, as well as other factors including the availability of caffeine, have altered human sleep patterns from what is chronobiologically optimal.

Several disorders have been identified as the result of deviations from a normal sleep-wake cycle, such as shift work, which is associated with a severely increased risk of premature death, as well as many other negative health outcomes.^3^ A novel approach is necessary to minimize the damage from altered sleep-wake cycles because the damage caused by these disruptions, in terms of healthcare costs, is extremely significant.

In addition to the effects on sleep-wake cycles engendered by the adoption of electrical lighting, what goes unappreciated is the biological effect of the light exposure itself. Exposure to non-native blue light is associated with a drop in melatonin production, and subsequently, sleep disturbances, including insomnia. Blue light exposure at night is also associated with a risk of depression, as supported by strong mechanistic data.

Light in the educational context represents both a current problem and an opportunity. The school environment, being a significant part of the pupil’s day, plays a significant role in the growth and development of the child, both in terms of biology and general health, as well as in terms of educational and life outcomes. Long-term learning is neurologically disturbed when deep sleep is interrupted, and many pupils are thought not to be getting enough deep sleep.^4^ Bucklo et al.^5^ found that direct current (DC) (electrical signals) in neurons altered their signal direction (traveled in reverse) during sleep in comparison to wakefulness. The same result was found with the DC changing polarity in the interfacial water layer below the myelin.^6^ Spano et al.^7^ found that sufficient sleep was necessary to prepare synapses for learning. Exposure to artificial light at night (ALAN) interferes with this process.

The widespread adoption of electric light is heralded as enabling greater access to education because pupils are no longer limited to daylight hours, when in less developed regions they are often occupied with work to support their families, for reading and writing. For this reason, the electrification of schools is identified as a United Nations (UN) Sustainable Development Goal (SDG).^8^ As such, the wide availability of electric light has made education more accessible to millions of children. However, as with the broader adoption of electric light, there have been some unintended biological, social, and environmental consequences.^9^ While in general children and adults show similar biological responses, the light environment of children warrants special attention, owing to their active growth and development as well as their need for learning.

Improved lighting has been associated with improved educational outcomes in several studies, and this has been largely attributed to its effects on vision and alertness.^10^ We propose that there are yet unappreciated biological mechanisms underlying this effect and that the pupils light environment could be improved through cost-effective interventions.

### Ecological Impacts of Artificial Light

In recent years, there has been a growing movement to provide energy-saving light sources for public lighting, with extensive applications in offices, schools, hospitals, etc. However, the possible effect of artificial light to decrease biodiversity in comparative biology and bioconservation^11–13^ has spurred the need to evaluate its effects on the abundance and survival of biosystems. The effects of artificial light have been reported on plant and animal physiology, behavior, reproduction, etc,^14–16^ with significant evidence for the effects of natural lighting on growth enhancement, circadian rhythms, pollination, and so on of plants.^17–19^

A common phenomenon is the attraction of insect communities, especially moths, to artificial light sources at night, leading to an extensive reduction of populations,^20^ dysregulated sexual activity, and disturbed reproductive cycles.^21^ In addition to moths, the mating behavior of fireflies is affected,^22^ as is the ability of dung beetles to navigate by starlight, forcing them to navigate by terrestrial cues.^23^ Photonreception in living systems is made possible through specially differentiated cells and organelles with sensitivity to specific wavelengths. In plants and some unicellular organisms, the molecules called phytochromes display maximal sensitivity in the red wavelength range, which allows light to activate the phytochrome HIS-kinase activity. Phytochromes also play a synergistic role with the blue light photonreceptors (cryptochromes) to allow photondetection and optimal functioning of the machinery of circadian rhythms in biological systems. Any disturbance in time-measurement circadian systems could have serious consequences for diverse normal physiological processes, which critically depend on circadian rhythms.^24, 25^ Cryptochromes are members of the photolyase family of proteins, which operate between 350 and 530 nm and are involved in DNA repair, phototropin expression, magnetoreception, orientation, and etc.^26^ Although specific to land plants and green algae, phototropins are sensitive to blue and UV-A light and are involved in the accumulation of chloroplasts, opening of the stomata, stem bending, and leaf positioning.^27^

### Human Health Impacts of Artificial Light

**Figure 2.**
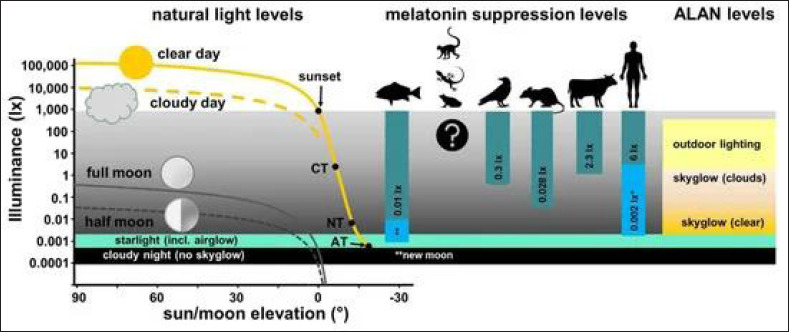
Reproduced with Permission from Grubisic et al.^33^ Minimum Levels Reported in the Literature to Suppress Melatonin (MEL) in Vertebrate Groups Relative to Light Levels by Natural and Artificial Light (ALAN) Sources.

**Note:** Left panel: illuminance during day, twilight, and night as a function of elevation angle of sun and moon; the shaded gray bar symbolizes twilight (CT—end of civil twilight, NT—end of nautical twilight, AT—end of astronomical twilight), the light-green bar symbolizes clear sky illuminance, and the black bar symbolizes illuminance during overcast night; yellow solid line—sun illuminance on clear day, yellow dashed line—sun illuminance on cloudy day, gray solid line—moonlight full moon, gray dashed line—moonlight first and last quarter (“half-moon”). Central panel: minimum melatonin suppression levels published in the reviewed studies for different vertebrate groups: fishes, amphibians, reptiles, birds, rodents, ungulates, non-human primates, and humans. Due to the low number of studies (*n* = 2), the levels reported for non-human primates are not shown. Right panel: typical illuminance levels from ALAN; *indicates minimum level of monochromatic light (460 nm) that suppressed MEL in controlled laboratory conditions,^34^ **indicates a significant suppression of MEL at new moon conditions, probably in the range of a few millilux, but light intensities were not measured in this study.^35^ Icons were made with Freepik (www.flaticon.com). Reproduced under a Creative Commons Attribution 4.0 International License (http://creativecommons.org/licenses/by/4.0/).

Although it has been realized that increasing light pollution is caused by uncontrolled development and urbanization, its critical role in human health and core biological processes has not been fully appreciated. Light pollution could have far more damaging biological effects than ever imagined, especially in learning environments employing intensive lighting systems during the day as well as the nighttime for teaching and learning activities. One of the many abnormal processes caused by artificial light at night time is suppression of melatonin production, which is crucial for initiating and maintaining sleep, in addition to other vital physiological functions such as immune response, antioxidant defences, haemostasis and glucose regulation.^28^ The relay of information on the stage of the light-dark cycle in humans is carried out by non-retinal photonreceptors in the eye to the suprachiasmatic nucleus and then to the pineal gland in the brain, which governs the secretion of melatonin (Figure 2). Melatonin is programed to start at sunrise and build up during the day to be released at sunset.

Exposure to artificial light sources at night increases the risk of melatonin suppression, which has been reported in several studies with a high likelihood of nocturnal melatonin suppression at moderate to high exposure settings.^25, 29, 30^ The epigenetic remodeling caused by artificial light at night (ALAN), leading to suppressed melatonin levels, has been linked to cancer risks in humans and increased mortality in animals attracted to light sources at night.^31, 32^

The cognitive effects of light have been investigated in several studies.^36^ In particular, most of these studies have focused on patients with disorders, in particular Parkinson’s disease, Alzheimer’s disease, traumatic brain injury (TBI), and others. The use of transcranial laser stimulation, also known as photonbiomodulation (PBM), has been observed to improve cerebral oxygenation^37^ and boost cognitive performance,^38, 39^ including positive effects on emotional regulation^38^ and executive function.^40^ Positive effects on memory were also observed for adults with mild cognitive impairment,^41^ and reduced the effect of PTSD on memory in a mouse model.^42^ Laser therapy has also been observed to improve symptoms of depression,^43, 44^ anxiety,^44^ and TBI.^45–48^

There is additional evidence of the ability of pulsed, near-infrared PBM to modulate neural oscillations,^49^ suggesting the possibility of entrainment, which may be used to improve learning given that alterations in brainwaves have been correlated with explicit and implicit learning.^50^ While some of these studies make use of individualized PBM devices applied to the head, there is also a marked influence of ambient lighting on cognitive health. Exposure to bright light in the morning improved symptoms of agitation in Alzheimer’s disease or related dementia (ADRD) patients,^51–54^ as well as motor restlessness.^55^ The impact of light on ADRD patients was covered in a 2013 review by Hanford and Figueiro.^56^ The wider effects of light on cognition and executive function were reviewed in a recent 2021 article by Siraji et al.^57^

### Biological Mechanisms

**Figure 3.**
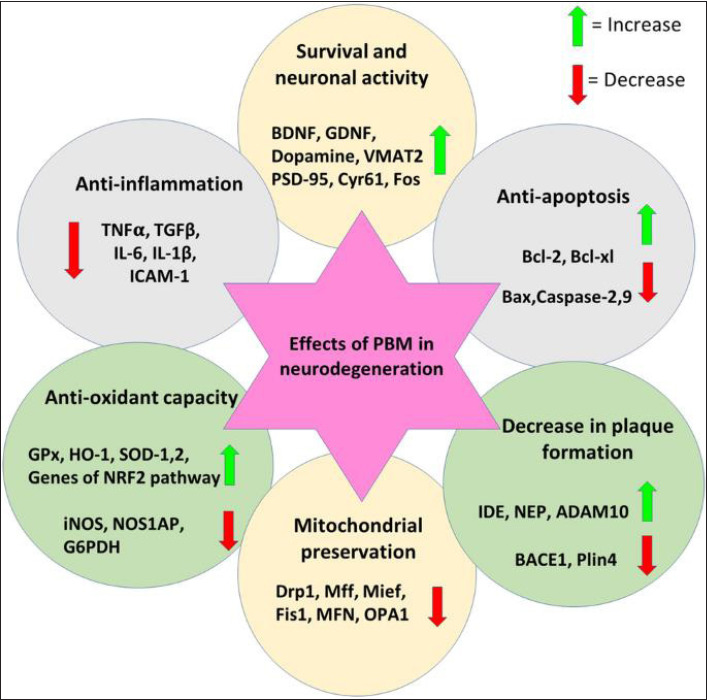
Reproduced with Permission from Bathini, Raghushaker, and Mahato.^64^ Overall Effects of PBM on Genes and Proteins Playing Different Roles in Neurodegeneration.

**Note:** Reproduced under a Creative Commons Attribution 4.0 International License (http://creativecommons.org/licenses/by/4.0/).

Artificial light interferes with normal physiological function through several mechanisms, including circadian dysregulation (Figure 3). In the human eye, blue light significantly lowers choroidal thickness and axial length by several folds when compared to red light, broad-spectrum light, and darkness.^58^ This results in significant changes in the non-image-forming neural pathways involved in brain myelination, growth, and development.^59^ While blue light can light an indoor space in an energy-efficient manner, important biological signals are omitted when pupils are exposed to a reduced spectrum. The far ends of the visible light spectrum have important biological effects, even though photon counts at those energies are significantly lower than those in the visible light section of the spectrum.^60^ At shorter wavelengths, UV photons are important for the synthesis of vitamin D.^61^ At longer wavelengths, far-infrared (IR) photon exposure from a low-frequency pulsed IR laser was associated with accelerated tissue regeneration after injury, likely through the TRPV1 pathway.^62^

PBM acts through several mechanisms; in the case of neurodegeneration, PBM lowers inflammation,^63^ increases neurogenesis through stimulating brain derived neurotrophic factor (BDNF), reduces apoptosis (programed cell death), lowers amyloid plaque formation, preserves mitochondrial function, and increases anti-oxidant activity.^64^ At a molecular level, PBM stimulates the mitochondrial electron transport chain (ETC), increasing the amount of energy available to neurons.

While in most of these studies the light is applied transcranially, however, this may not be feasible in a normal classroom setting as it usually requires a specialized helmet setup. There is some evidence for the remote effects of PBM, where the light is incident on a different part of the body.^65, 66^ After light treatment, increased blood flow is observed^67, 68^ and increased blood flow is a marker of improved cellular activity.

### Interventions

The proposed adaptations to current schools can be broken down in terms of intervention type (social, architectural, etc.), cost regimes, or biological mechanisms modulated. We will first explore basic improvements, which require only small changes to achieve a positive effect (Table 1).

**Table 1.** Possible Interventions to Improve Lighting for Students and Schools.

Intervention	Implementation	Cost	Effect	Biological Mechanism
Blue light filter on electronic devices	Install app on electronic devices	Free	Improves sleep	Decreases suppression of melatonin
Red light spectrum at night	Cover sources of ALAN with red colored tape	~EUR 5 per roll	Improves sleep	Decreases suppression of melatonin
	Use candles or red light for lighting in late evening	~EUR 6 per 100 pack of candles or ~EUR 3 per red bicycle light		
Light exposure in morning	Pupil spends first hours of sunrise in direct sunlight	Free	Improves concentration and learning	Priming circadian rhythm

One such intervention is using red light for instruction in the evening in order to foster better sleep by not suppressing melatonin production in the late hours (Table 1).

Additionally, photontherapy in the form of light exposure, either natural or, if conditions do not allow, an artificial alternative, could greatly improve children’s growth, development, and learning. Where possible, outdoor classrooms should be preferred over indoor instruction. To enable improved access to outdoor classrooms, developments in presentation technology visible in daylight could contribute to this shift. Additionally, shifting to outdoor instruction would also herald a shift towards more hands-on, experiential learning, which, while outside the scope of this review, has been demonstrated to be superior to standard classroom pedagogy by many metrics.

Practically, the switch to red LED bulbs over fluorescent tubes and energy-efficient light bulbs produces cost savings over the life of the bulb, so any initial investment is recovered over the life of the bulb. The use of dynamic lighting, where the lighting changes throughout the day and for specific tasks, could have beneficial effects for student learning.^10, 69^

One intervention involves swapping glass windows for plexiglass, which allows for greater UV penetration and could have positive health benefits for the pupils. Furthermore, other architectural modifications that increase natural light exposure, such as large, sun-facing windows, sunroofs, and dedicated solariums, could also be created to improve the student experience. When building new schools, maximizing natural light exposure is a crucial factor in designing the architecture of the buildings.

With natural and UV light, public health messaging has significantly emphasized the dangers of UV exposure without due consideration to its benefits,^70^ which include vitamin D synthesis and a lower incidence of many diseases such as autoimmune disorders and cancer.^71^ Public health messaging has largely focused on the risks of UV light, encouraging people to avoid UV exposure. This approach has come into question,^72^ and recent studies show avoidance of sun exposure is linked to higher all-cause mortality.^73^

Other challenges toward the adoption of these changes may be cultural in nature, emerging from the teaching disciplines themselves, concerning classroom management and discipline issues.^74, 75^ Teachers who are successfully applying outdoor learning report marked improvements in the educational experience, including improved student–teacher relationships, the salience of lessons, and a more relaxed atmosphere.^75^

More broadly, these changes could herald an era of greater integration of the natural world into the built environment. There has been a broader trend for this incorporation leading to a synthesis between natural and built elements, for example, the inclusion of student-maintained gardens into primary and secondary education schools. Not only does this teach children practical skills, such as how to grow foods, but it also fosters an appreciation for the natural world, and participating students have reported greater interest in their food following this experience.^76^ This field is often termed ‘biophilic design’, borrowing a term coined by Edward O. Wilson.^77^ Wider adoption of biophilic design represents a positive approach for directly addressing ecological issues and, by inducing ecological awareness in students, reminding them that they are part of the natural world.^78^ More contact with nature has many proven benefits for children,^79^ including cognitive benefits.^80^

The principles of biophilic design have been covered in some reviews,^81^ including articles specifically focused on primary schools^82^ and universities.^83^ Different building techniques, including solarium designs, windows with improved light penetration, both in terms of surface features and orientation, as well as “light pipes,”^84^ can increase pupils’ exposure to sunlight. Increased sunlight exposure has been associated with higher test scores,^85^ increased productivity,^86^ and better mood in students.^87^ Biophilic architectural design can overcome many issues with the current built environment and promote better health.

Electro-biomodulation (EBM) through nature’s contact also has positive health impacts if pupils can be in bare and or ESD foot/hand contact with the Earth. The clinical benefits have been demonstrated on inflammation^88^ and blood flow,^89^ wound healing and rehabilitation,^90^ increased redox stabilization in the presence of external noise-signal disruptors,^91^ muscle and physical stress loading,^92–94^ hypertension,^95^ and vagal nerve tone, which is associated with stress resilience.^96^

For the case of digital education, which has greatly expanded in the wake of the coronavirus outbreak, termed a pandemic by the World Health Organization in March 2020, specific adaptations can be made to improve educational and health outcomes. Hybrid learning is likely to become standard in years to come, enabling greater accessibility while also presenting its own challenges and opportunities for student health.

One such challenge of hybrid or hyflex models involving asynchronous classes (or synchronous classes from another time zone) is that students may be using digital devices late into the night. For digital classes, students should be encouraged to download effective blue-light filters on their devices,^97^ which can help to minimize sleep disruption due to the melatonin-suppressing impacts of blue light.^98^ Students are also encouraged to expose themselves to bright light early in the morning, ideally sunlight, in order to improve their mood and alertness.^99^

### Economic Benefits

Education is associated with many improved life outcomes for pupils, including better health, higher earnings, and greater life satisfaction.^100^ Improved education is also associated with greater economic growth and prosperity in society.^101^ If these inexpensive interventions (Table 1) can raise human capital by only a few percent, then they will easily pay for themselves.

We summarize this literature to make conservative estimates for the effect of changes in lighting based on intervention studies performed. The possible magnitude of the effect in the literature is modest but significant. One issue that educators face is the post-lunch dip in performance, which can be alleviated by exposure to bright light.^102^ There have been few studies considering the biological mechanisms covered in this article, and most focus on alertness.99 Nonetheless, the health benefits are difficult to predict, but intervention studies in other contexts, including the home environment for elderly people, show improvements in deficits caused by aging.^103^

While a rigorous assessment of the benefits of changes in lighting requires a meta-analysis to accurately determine the effect size, these interventions are inexpensive enough to implement, and even a slight increase in pupil’s learning would make this a worthwhile investment.

## Conclusion

In conclusion, light has multifactorial impacts on the health and well-being of students, and the prevalence of artificial light as well as the decreased exposure to natural light have created challenges for their health and well-being. The growing prevalence of electric lighting has brought with it ecological and health challenges, which could be allayed by educators and designers through better design of the indoor light environment.

Light affects student health and well-being, as well as fundamental learning outcomes through multifarious mechanisms, including the regulation of circadian rhythms. First and most obviously, it allows the student to see images for verbal learning and remain alert throughout the day. Second, there are effects on the circadian and hormonal health status, and there are also brain-specific effects of light exposure.

Several interventions can be used to improve the light-related factors affecting student health. These include primarily the encouragement of more outdoor learning experiences and teaching about the importance of light for health in classrooms. Second, classroom and school design can incorporate design features like UV-penetrating glass, larger windows with better sun orientation (as opposed to orienting windows based on Cartesian rectilinear design), and sun pipes to increase the exposure of students to natural light.

Schools can also convert their bulbs to emit a broader spectrum of light, better approximating the wide spectrum of natural sunlight. Certain fluorescent tubes with a limited and cold (i.e., blue) spectrum, along with a high flicker rate, can cause attention problems in children as well as decreased cognitive performance. These lights should be avoided in favor of broader-spectrum lighting. Simultaneously, students, especially hybrid or virtual students, may be educated about the effects of blue light by installing one of many blue light screen filters on their electronic devices calibrated to a photon-flux model.

Finally, these interventions, though requiring minimal investment and foresight, could potentially have wide-reaching benefits for students, both in terms of improved health and life satisfaction and in terms of learning outcomes. Improving the lighting environment for students is well worth it from a financial and public health perspective.
